# Transforming students into digital academics: a challenge at both the individual and the institutional level

**DOI:** 10.1186/s12909-015-0330-5

**Published:** 2015-03-14

**Authors:** Maria Thorell, Peter Kindt Fridorff-Jens, Pia Lassen, Theis Lange, Lars Kayser

**Affiliations:** 1 Centre for Online and Blended Learning, Faculty Administration, University of Copenhagen, Blegdamsvej 3 B, 2200 København N, Denmark; 2 Center of Evaluation, Faculty Administration, University of Copenhagen, Blegdamsvej 3 B, 2200 København N, Denmark; 3 Department of Public Health, Section of Biostatistics, University of Copenhagen, Øster Farimagsgade 5, 1014 Copenhagen, Denmark; 4 Department of Public Health, Section of Social Medicine, University of Copenhagen, Øster Farimagsgade 5, 1014 Copenhagen, Denmark

**Keywords:** Students, University, ICT, Digital literacy, Alignment, Online behaviour

## Abstract

**Background:**

Little is known of students’ Information and Communication Technology (ICT) readiness in a learning context. Information about students’ capabilities and resources is an important prerequisite for designing meaningful teaching and learning activities that engage and motivate students. To learn about health science students’ usage of digital equipment, familiarity with software, online behavior and communication with the university, we have conducted a survey focusing on these areas.

**Methods:**

A digital questionnaire was sent to 9134 health science students, of whom 1165 responded (12.8%).

**Results:**

Almost all students owned a laptop (98.3%) and a smartphone (86.5%) and used these for internet access. The students were most familiar with typical office programs like word processing and spread sheets. Students used social media in their private lives but to a lesser extent in relation to their studies; they also experienced that their teachers made limited use of these media. The most commonly used tool for working with fellow students was email (80%) and for communication, SMS (47.6%). An age difference was found in relation to the way students communicated with each other. The mean age of chat users was 23.8 (Standard deviation 3.7) years, SMS users, 25 (Standard deviation 4.2) years and email users, 27.9 (Standard deviation 6.5) years. Over half of the students (53.4%) found that the degree of ICT incorporated in the teaching and learning activities was insufficient to provide them with the skills necessary in their future profession.

**Conclusions:**

Although a large percentage of the students had access to the internet, reported familiarity with basic software and used online services in their private lives, they were unfamiliar with the software and services they were expected to use in their studies. The students experienced that teachers did not use internet resources, which apparently influenced their perception of the importance of, and thereby their usage of, these services. The way the younger students communicate differs from the way communication takes place at the university, and it is recommended that the institutions should look into how they can meet the students in ways they are familiar with.

**Electronic supplementary material:**

The online version of this article (doi:10.1186/s12909-015-0330-5) contains supplementary material, which is available to authorized users.

## Background

The expansion of smartphones and internet access along with the increasing use of social media and free online courses and learning materials are revolutionizing the way students learn, communicate and collaborate. It is challenging the role of universities in both their ICT infrastructure and the way they communicate and design learning activities. Very little is known of students’ readiness for using ICT in the context of learning. We have identified a few dated studies addressing the use of ICT in education, but these studies relate to specific courses or situations, for example, the use of the internet or smartphones [1-3], and cannot be used to draw general conclusions about students’ ICT competencies and readiness. In a UK study investigating the use of smartphones in 2012 it was found that 59% of university students used smartphones [4], which was lower than the percentage reported for medical students in Canada in 2011 (85%) [5]. One report from 2013 describes the use of social media and the internet in general among international students. Here it was found that social media were used by most students and laptops were the most widely used devices to access the internet [6]. A previous study from the Faculty of Health and Medical Sciences at the University of Copenhagen (UCPH) has demonstrated that students’ motivation to use digital services, such as a histology database, is related to how teachers integrate these services into their courses [7]. This demonstrates the importance of the teaching and learning context.

Teachers’ digital readiness may influence how they design educational activities in a way that engages and motivates the students. It might be assumed that since teachers generally belong to an older generation than the students, they may be considered immigrants to and not natives of the digital universe. Yet a study from Aarhus University, Denmark [8] has shown that while there are differences they may not be so great that they create a gap between teachers and students; the authors of that study recommend pursuing the investigation further.

When designing curricula and courses, it is important to know what can be expected from students studying in traditional classroom environments with respect to capabilities and resources. This information is also an important prerequisite for designing meaningful teaching and learning activities that engage and motivate students without imposing digital barriers or appearing too simplistic.

Most of the current students at UCPH were born in the late 80s and early 90s, and were therefore raised in the digital society. For them, there has been no transition phase, unlike older generations, who recall adapting to the “new media”.

The new active and involving use of media arises from the groups of youngsters that Oblinger and Oblinger calls the Net Generation [9]. Born in the late 1980s and onward, the Net Generation has been raised under entirely different technological conditions. The Net Generation is born into an information society, using digital media in every aspect of their lives from information seeking to communication and socializing. Compared to older generations, the Net Generation has a different but natural approach to technology, and they use digital media for knowledge construction and learning [9].

Another way to understand these differences between the younger generation and the older generations is introduced by Prensky who uses the terms “digital natives” and “digital immigrants” [10]. Immigrants must build skills and acquire knowledge of digital media, whereas the natives use it as a natural part of living. According to Oblinger and Oblinger [9] and Prensky the university students belonging to the Net Generation are considered to be digital natives and are expected to easily use technology as an integrated part of their everyday lives. The authors’ observations (PKJ, MT, LK) have raised doubts about whether they really are. In order to develop effective and efficient educational initiatives that will evolve and motivate the students, it is important to explore whether the students are natives or whether a digital divide exists in this generation.

In this study, we combine the perspective of the students with respect to their personal resources and digital behaviour with their experience of how faculty and administrative staff meet them. This will help develop an understanding of how the digital intersection between students and the institution may need to be aligned to accommodate the students’ needs and competencies and to point out where higher education institutions may change their teaching and learning activities.

Our questionnaire will be available in English and can be used for comparative studies around Europe and in other regions.

## Methods

A survey was constructed to explore equipment, self-reported IT skills and online behaviour among students at the Faculty of Health and Medical Sciences. The questionnaire consists of 15 questions covering the following topics:

- personal digital equipment
- familiarity with software
- online behaviour
- alignment of expectations with students with respect to communication and tasks

The questionnaire was constructed on the basis of a brainstorm and a brief description of intended indicators. A first draft was evaluated after interviewing students about their understanding of the meaning of the questions. Then a second draft was written and tested with a small group of students. This version was again rewritten and then entered into an electronic questionnaire which was again tested with a third group of students. The questionnaire was translated into English by the authors and after testing updated to reflect the final Danish version. The translation of the English questionnaire was validated by a bilingual native English speaking Canadian.

The questionnaire was distributed to all students at the Faculty three weeks after the Fall Semester of 2013 started. This time of year was chosen as students were not being asked to complete other university surveys (for example, course evaluations) at the same time and as the newly admitted students had had time to gain some experience with the university systems. The students received an email from the Vice-Dean for Education, asking them to fill in the survey. The email contained two links; one to the Danish version and one to the English version for the international students. After one and two week’s reminders were sent to non-respondents and after three weeks the survey was closed. The students were also informed and asked to fill in the survey through the Faculty information system on electronic screens placed around campus and on the intranet.

The survey was send to all Bachelor’s and Master’s Students, both full-time and part- time: in total, 9134 students. Sixty-three emails bounced due to administrative errors. A total of 1165 (12.8%) students answered the survey fully or partially. For background data see Table 1.

**Table 1:** **Distribution and characteristics of students on educations at the Faculty of Health and Medical Sciences**

Study program	Total students	Number of respondents (N = answered fully (answered partially))	Response rate	Age, mean	Age, std dev	Gender, F	Gender, M
**Bachelor**							
Medicine	1741	261 (8)	15.0%	23,3	3,9	59,5%	40,5%
Pharmacy	788	87 (15)	11.0%	22,2	3,5	77,5%	22,5%
Veterinary Medicine	597	94 (3)	15.7%	23,8	5,1	88,7%	11,3%
Biomedical Engineering	332	7 (2)	2.1%	24,0	2,7	33,3%	66,7%
Dentistry	297	46 (3)	15.5%	24,3	6,4	81,6%	18,4%
Public Health	205	25	12.2%	22,6	3,5	92,0%	8,0%
Dental Hygienist	197	23 (2)	11.7%	24,8	5,1	88,0%	12,0%
Molecular Biomedicine	165	23 (1)	13.9%	22,1	1,9	50,0%	50,0%
Health Informatics	156	24	15.4%	25,3	10,0	45,8%	54,2%
**Master**							
Medicine	2009	271 (10)	13.5%	26,8	3,3	59,8%	40,2%
Veterinary Medicine	533	68 (4)	12.8%	26,5	3,2	91,7%	8,3%
Pharmacy	412	47 (1)	11.4%	24,9	1,9	81,3%	18,8%
Public Health	208	24	11.5%	25,7	1,4	87,5%	12,5%
Dentistry	178	15 (4)	8.4%	25,1	1,4	78,9%	21,1%
Molecular Biomedicine	133	14	10.5%	25,0	1,3	78,6%	21,4%
Pharmaceutical Sciences (specialization in medical sciences)	122	13 (1)	10.7%	25,8	2,4	78,6%	21,4%
Human Biology	107	9 (2)	8.4%	25,4	1,5	72,7%	27,3%
Health Science	92	13	14.1%	32,3	3,5	76,9%	23,1%
Environmental Chemistry and Health	56	2	3.6%	24,1	0,2	100,0%	0,0%
Global Health	35	5 (1)	14.3%	24,6	0,7	66,7%	33,3%
Health Informatics	32	8	25.0%	25,5	3,4	62,5%	37,5%
Pharmaceutical Sciences (specialization in pharmaceutical Sciences)	31	3	9.7%	26,6	0,8	33,3%	66,7%
Medical Chemistry	17	5 (1)	29.4%	24,5	0,9	33,3%	66,7%
**Part-time master**							
Public Health	71	4	5.6%	44,8	7,0	100,0%	0,0%
Master of Disaster	59	9	15.3%	31,1	3,3	33,3%	66,7%
International Health	38	7	18.4%	33,5	9,1	42,9%	57,1%

For each education the tables lists the number of respondents, response rate, average age and standard deviation of age in years and the frequency of females and males given in percent.

### Ethics

In this study, no biological material or medical devices were used and the participants were not subjected to any kind of diagnostics or treatment. Consequently, approval from the Danish National Committee on Health Research Ethics (*Den Nationale Videnskabsetiske Komite*), was not required, which is the case for all studies only involving interviews and questionnaires [11].

This was confirmed by presenting the protocol to the Regional Ethical Committee (protocol number VEK H-2-2013-FSP64). Data was anonymous and handled according to the Danish Data Protection Law; therefore a registration with the Danish Data Protection Agency was not required.

### Statistical analyses

Descriptive statistics are presented as mean values with standard deviations (Std dev) for numeric data and percentages for categorical data. Categorical outcomes (e.g. the use of smartphones) are compared using Chi-sq tests (Pearson) and numerical responses (e.g. age) are compared using t-tests. Association between numeric variables is expressed by linear regression. A significance level of 5% was employed throughout. Statistics were analysed using SData version 6.

## Results

### Personal digital equipment

A considerable number of students have a smartphone with the most common operative systems installed (Table 2). Part-time Master’s students differ in their preference for smartphones, as only 79.5% have one (confidence interval: 72.1-87.9%); 50.7% have an android-based phone in contrast to full-time students, of whom only 35% have an android-based phone. Female students have a higher usage of smartphones (88.2%) compared to male students (83.1%) (p = 0.019); there is no age difference (p = 0.47). Almost all students have a laptop and only one-fifth of the students have a stationary PC. The operating systems were primarily Windows and IOS.

**Table 2:** **Students’ personal digital equipment categorized by device type and operating systems**

	Do have	Apple OS	Android	Windows	Linux	Other	Do not have
Smartphone	86.5%	61.3%	36.4%	2.3%	-	1.3%	0.2%
None-smartphone	13.3%	-	-	-	-	-	
Stationary PC	18.3%	16.2%	-	82.9%	1.0%	0.0%	81.7%
Laptop	98.3%	41.2%	-	57.9%	0.9%	0.0%	1.7%
Tablet	28.0%	78.0%	15.2%	6.8%	-	0.0%	72.0%

Only 77.6% report accessing the internet with their smartphones; 91.3% report doing so with their laptops. One quarter of the laptops have a battery lifetime of more than six hours. Forty-three percent of the laptops have a battery lifetime of less than four hours.

### Familiarity with software

Sixty-nine percent of all students reported that they were familiar with apps (scores four or five on a five-point Likert scale). Sixty-nine percent of the smartphone users have downloaded at least one new app in the last month; 77% hardly ever get help from others with download, as opposed to 8% that almost always get help from others. As for settings, 75% never get help from others and 6% always get help from others with settings. There appeared to be no correlation between age and familiarity with apps (p = 0.274).

Most students are familiar with word processing while fewer are familiar with spreadsheets (Table 3). There is a high negative correlation between age and familiarity with word processing (linear regression coeff. = −1.62; p < 0.0001), demonstrating that the younger students were most familiar with the software. In contrast, no significant correlation is seen between the level of study and familiarity with word processing (p = 0.63). There is a weak negative correlation between familiarity with spreadsheets and age (linear regression coeff. = −0.3; p < 0.05).

**Table 3:** **Familiarity with software and redirection of university emails received in the university portal to a personal email account**

	Not at all (1)	(2)	Somewhat (3)	(4)	Very (5)
Word processing (e.g. Word)	0.2%	0.3%	3.2%	20.7%	75.7%
Spread sheet (e.g. Excel)	3.1%	8.6%	29.3%	27.1%	31.9%
Apps for smartphones	8.2%	5.5%	16.9%	23.3%	46.1%
Reference management software	41.1%	13.6%	21.9%	14.9%	8.5%
Redirection of UCPH mail	29.3%	16.2%	20.8%	13.9%	19.8%

Only one quarter of all students are familiar with reference management software (Table 3). Interestingly in the Master programs the familiarity of reference programs was higher ranging up to respectively 44% for medicine and 42% for public health. Also 45% of part-time Master’s students reported that they were familiar with reference management software. These observations are supported by a positive correlation between familiarity with reference software and age (linear regression coeff. = 0.55; p < 0.0001).

Furthermore 69% of all students reported that they never used advanced software (e.g. virtual microscopy or Chemdraw) in connection with their studies. Male students used advanced software slightly more often than female students (Mean 1.6 vs. 1.2 (Std dev 1.8 vs. 1.6) (7-point Likert scale), p < 0.0001; see Additional file 1, Q5). Conversely, no significant correlation is seen between age and the use of advanced IT programs (p = 0.12).

### Online behaviour

Students have multiple search options at their disposal when searching for information in relation to their assignments; a large percentage (86%) use non-academic wide search engines such as Google, whereas a small percentage (27%) use narrow search engines such as Google Scholar. More than half of the students use more dedicated academic services such as PubMed (65%) and library search engines (55%).

In relation to their studies, 53% of the students use social networks such as Facebook on a daily basis, whereas only 1% use professional networks and microblogging such as LinkedIn and Twitter. Seen from another point of view the percentages of students never using social networks, professional networks and microblogging are 6%, 61% and 76% respectively. Furthermore, 16% use multimedia services such as YouTube and Instagram on a daily basis, while 19% reported that they never used them.

Male students had a lower use of social networks compared to female students. (Mean 4.5 vs. 4.9 (Std dev 1.9 vs. 1.8) (seven-point Likert scale) p < 0.01; see Additional file 1, Q5). In contrast, male students had a higher use of microblogging (Mean 0.6 vs. 0.4 (Std dev 1.3 vs. 1.0) (seven-point Likert scale) p = 0.01; see Additional file 1, Q5), and multimedia services (Mean 3.2 vs. 2.8 (Std dev 2.1 vs. 2.1) (seven-point Likert scale), p < 0.01; see Additional file 1, Q5). There is no gender difference in the use of professional networks (P = 0.24).

### Online materials

Male students refer to online materials more often than their female peers (Mean 2.8 vs. 2.5 (Std dev 2.0 vs. 2.0) (seven point likert scale) p < 0.01; see Additional file 1, Q5).

There was a negative correlation between age and use of social networks (−0.69; p < 0.0001) and a positive correlation between age and use of professional networks (0.64; p < 0.0001). There was a weak positive correlation between age and use of microblogging (0.42; p = 0.001) and no correlation between age and use of multimedia services. There was a weak positive correlation between age and referral to online material (0.34; p < 0.001).

**Figure 1:**
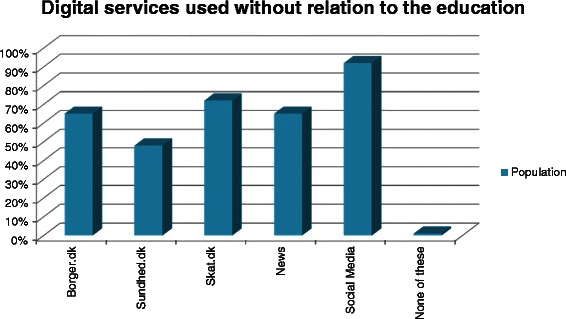
**Digital services used without relation to the education.** Usage of digital services offered by the government, are commonly used by citizens in Denmark. Borger.dk is the general citizen platform offered by the government and municipalities, sundhed.dk is a health care portal and skat.dk is the tax registration platform. News is national and international media e.g. New York Times, the Sun and eb.dk. Social media covers social networks and multimedia platforms e.g. Facebook, LinkedIn, Instagram and YouTube. The usage is given as percentage of students who use each of these services.

Outside their studies, students use the digital services offered by the Danish government and regions, such as the tax reporting platform, skat.dk (72.3%), the municipalities’ communication platform citizen.dk, borger.dk (64.2%) and the health portal, sundhed.dk (47.9%) (Figure 1). A total of 78.6% hardly ever use professional networks and 92.6% never use microblogs. The age of users compared to those not using the services was higher for health portal (Mean 25.6 vs. 24.3 (Std dev 4.8 vs. 4.3); p < 0.0001) and news media (Mean 25.2 years vs. 24.3 years (Std dev 4.7 vs. 4.4); p < 0.01), whereas the age of users of social media was lower (Mean 24.7 years vs. 26.8 years (Std dev 4.3 vs. 7); p < 0.0001).

A higher percentage of male students use news media outside their studies than expected (chi2 = 20.889; p < 0.0001), whereas there is no gender difference for other services such as the citizen, health and tax portals as well as social media.

### Alignment of expectations

#### Communication

**Figure 2:**
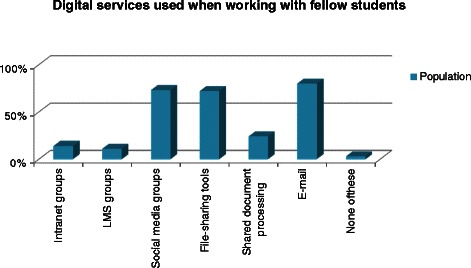
**Students’ usage of digital services when working with fellow students.** Usage of digital services for collaboration with fellow students. Intranet groups are offered in the university’s Sharepoint intranet for all students and employees. LMS groups are offered in the learning management system aimed for collaborative work between students and with the teachers. Social media groups are offered by external providers such as Facebook and LinkedIn. File-sharing tools are e.g. Dropbox and Google Drive aimed at only sharing files such as documents and spread sheets whereas shared document processing tools are services provided for collaborative and synchronic work, e.g. Google Docs. Email is used for asynchronous communication and document exchange between the students. The usage is given as percentage of students who use each of these services.

There is a similar use of SMS and chat when communicating with fellow students; 47.6% prefer SMS and 38.0% prefer chatting. Very few students prefer email as a way of communicating with fellow students (14.4%). For study work with fellow students a combination of various digital platforms are used (Figure 2), i.e. 80% use email, 73% use groups in social media (e.g. Facebook or LinkedIn) and 72% use file-sharing tools such as Dropbox. For communication with fellow students, females have a higher preference than males for SMS (49% vs. 44%) and chat (39% vs. 36%), whereas males had a higher preference than females for emails (19% vs. 12%) (chi2 = 10.5769; p = 0.005), Additional file 1, Q9. The mean age of chat users is 23.8 (Std dev 3.7) years, of SMS users, 25 (Std dev 4.2) years and email users, 27.9 (Std dev 6.5). The youngest students (age group 18–22) use chat functions to a higher degree than email and SMS, whereas the group aged 22–27 has a higher use of SMS. The use of email tends to increase with age (chi2 = 124.2, p < 0.001).

#### Tasks

On the UCPH portal KUnet, students have access to the UCPH intranet, and thereby access to Self Service, the bulletin board, email and the UCPH learning management system (LMS), which is hosted by Itslearning Norway. The students are expected to use Self Service and email regularly, as email is the only form of written communication with the university. The students were asked how often they use the different services provided to them in the UCPH portal (Table 4); 78.5% answered that they use UCPH’s LMS several times a week while 3.7% hardly ever use it. There is a higher use of the LMS among medical students in the Bachelor’s program compared to the Master’s program (Mean 5.3 vs. 4.0 (Std dev 1.0 vs. 1.5) (seven-point Likert scale), p < 0.0001; see Additional file 1, Q4). With regard to UCPH email, 71.4% of the students use it on a regular basis as opposed to 4.0% who rarely use it and 21.3% who use it two-four times a month. Only 34% know how to forward their UCPH emails to an email account of their own choice. For redirection of UCPH mail, a low positive correlation between familiarity and age was found (linear regression coeff. = 0.25; p < 0.05) with older students being slightly more likely to redirect their emails than younger students. Additional services in the UCPH portal are rarely used; only 16% use services such as the bulletin board and SelfService on a regularly basis. When working with fellow students, 14% use group rooms in the UCPH portal (MS Sharepoint®) and only 11.0% use group rooms in the LMS.

**Table 4:** **Students’ use of digital key services for students at the university**

	Less than monthly	Once a month	Several times a month	Once a week	Several times a week	On a daily basis	Do not use
UCPH LMS	3.1%	3.0%	7.1%	7.8%	28.6%	49.9%	0.7%
UCPH mail	2.7%	3.2%	6.4%	15.0%	33.3%	38.1%	1.3%
UCPH portal’s other functions (e.g. bulletin board)	10.9%	24.5%	26.5%	19.2%	12.4%	3.6%	3.1%

Twenty-six percent of the students experience that their teachers use the LMS on a daily basis, whereas 38% experience that their teachers use the LMS several times a week. Only 3% find that the teachers do not use the LMS at all. Other services the students experienced teachers using once a month or less, are reference to online materials (73%), multimedia (80%), advanced software (84%) and social networks (90%).

Over half (53.4%) of the students believe that the IT incorporated in their studies is not sufficient to provide them with the necessary skills for their future profession.

## Discussion

Most of the students who participated in this survey may be considered to belong to the generation of digital natives. In accordance with this, most of the respondents had a laptop and a considerable number had a smartphone. However, as we discuss in the following, it is surprising that the students cannot be considered to be fully digitalized and also that the symbiosis of digital natives and a university with digital resources does not seem to have resulted in a student population with digital expertise.

### Personal digital equipment

The first prerequisite to create an effective and efficient digitally supported collaborative educational environment is that the students have digital mobile equipment and access to internet-based services when needed.

Nearly all students have laptops available and are able to use internet services either on their laptop or a smartphone. Therefore, students can reasonably be expected to use online material and participate in digital activities. This is already the case as all students are required to use the UCPH LMS and only communicate with the administration digitally. However, when it comes to the use of advanced learning technology, for example, apps, including clicker apps, the diverse landscape with students using two different operating systems on their computers and their smartphones imposes some challenges that the university must be aware of. It is difficult to plan to use smartphone-based activities as one sixth of the students do not have a smartphone, and of those who have a smartphone, one quarter needs assistance to some extent to download and configure new apps. Another issue is that although newer generations of laptops and tablets are expected to have battery lifetime up to 10 hours or more, almost half of the students’ laptops have a battery lifetime of less than four hours, which is not sufficient for a whole day of study activities. The university must provide power sockets in places where the students study and relax in order to ensure that they have sufficient access to digital services. The abundant use of laptops and smartphones for internet access is probably higher than the situation reported by Vanozzi & Bridgestock, who indicated that approximately 80% of students in Europe and 73% of students in the US are reported to access the internet primarily through their mobile devices [6]. In the present survey, students’ primary use was not addressed; instead, the question concerned whether or not the devices were used for internet access and the numbers are therefore not fully comparable. The number of smartphone owners among medical students was higher than that found in the UK in 2012 [4] but in accordance with the reported number for medical students in Canada in 2011 [5]. The lower number in the UK in 2012 may be due to a difference between digital readiness and financial capacity among the UK students [4].

### Familiarity with software

The second prerequisite for obtaining the benefits of a digitally supported educational environment is that the students are familiar with the software commonly and specifically used for academic work. Students enrolled at the university have completed high school, where they were expected to work with word processing and some calculation programs. At the university, the educational activities often require the use of word processing, spreadsheets and reference programs, e.g. for writing laboratory reports. It is noteworthy that although 96% are familiar with word processing and 59% are familiar with spreadsheets, only 23% are familiar with reference programs. The lack of familiarity with reference programs is a challenge for universities, as many students would benefit from using them. Often students are required to search for literature and manage citations when working on projects.

As students in the Master’s programs in Medicine and Public Health have a higher degree of familiarity with reference programs than students in the Bachelor’s programs, it may be expected that familiarity with reference programs generally increases over the course of their studies. However, the degree of familiarity is not satisfactory and it is surprising that digital natives do not use tools like these. Universities need to consider how educational activities can be planned systematically to develop academic digital competences.

### Online behaviour

The third necessary student competence is the ability to acquire and use online information. Most people are familiar with broad search engines such as Google, Yahoo and Bing. In accordance with this, more than 90% of the UCPH students use Google in relation to their studies. Interestingly, only 55-65% use dedicated academic services such as PubMed and library search engines. These services may be more relevant to the academic tasks that the students are expected to perform. In line with this, more than 90% of students use social media such as Facebook or YouTube in general, but only a little more than half of the students use social networks such as Facebook in relation to their studies. It may be due to lack of relevance for the tasks being performed, but it may also be due to the students’ perception of how digital services can be used academically in contrast to private use. Students’ use of social media outside of their studies is in accordance with Vanozzi & Bridgestock [6], where European students’ use of LinkedIn, Twitter, Facebook and YouTube ranged from 74% to 84%. Students at UCPH apparently have a lower use of microblogs such as Twitter and professional networks such as LinkedIn. Our study distinguishes between students’ use in relation to their studies and outside the studies and we have not been able to find other studies that make this distinction.

### Alignment of expectations

The three domains of the students’ personal equipment, familiarity with software and online behaviour are important prerequisites to understand the students’ competences. A fourth domain is how the university as an organization can contribute to an alignment of the students’ competencies and the services provided by the lecturers and the administration in order to create an educational environment that the students can benefit from. In this context it is important to understand how the students actually communicate and collaborate with each other and the lecturers and how they currently use the existing services provided by the university. On the basis of this knowledge it will be possible to suggest how the lecturers and administrative staff should address the students and how future services should be designed.

Our results show that a majority of students use email along with social networks and file sharing tools when working with fellow students. Interestingly, for communicating with fellows the students preferred SMS and chat rather than emails. This reflects the general change in society in how communication tools and services are used. According to the Danish Business Authority, the use of SMS has decreased by 13% in 2013, whereas the use of MMS has increased by 37% [12].

Our data reveal that the university has not adapted its services, approaches to teaching and learning and mind-set to the digital natives. Therefore, the university is not accommodating the students’ needs and preferences in communication, cooperation and ways of learning. The survey shows that a significant number of students use chat functions to communicate with student peers. As noted, UCPH has not adopted chat as a means of communicating with the students, or for that matter as a way for the students to communicate with the university. This form of communication stands in contrast to the rest of Danish society, which uses a more multichannel approach to information dissemination. Prensky highlights that natives are used to receiving information very quickly [10]; therefore, emails might be perceived negatively, as it may be frustrating to wait for a response.

It is difficult to keep up with the latest means of communication, but the study highlights the need for universities to be adaptable to new communication forms.

The digital natives work best in networks and require immediate feedback from lecturers They are used to working in parallel processes and multi-tasking and prefer visual rather than textual material [9,10]. According to Prensky, one of the biggest challenges in education today is that teachers speak an outdated language from the pre-digital age [10]. This becomes a problem when they teach a generation that speaks an entirely new language.

For instance, in typical university learning environments, information remains mainly text-based, possibly mediated via a PowerPoint presentation or an intranet, but the advantages of a multimedia based content delivery are rarely considered. This is a poor way of using modern media and can be seen as a parallel to using a computer and word processor like a typewriter.

The students’ relatively low familiarity with dedicated academic tools and lower use of social media in relation to their studies compared to outside their studies may be a result of a misalignment between students’ and teachers’ expectations of what is relevant to the learning process. When students experience that approximately 70% of their teachers do not use Facebook and that 50% do not use online material it is a signal that these services are not of relevance for the study program. The universities should consider whether social media may be relevant to incorporate in the study activities. One example is the use of LinkedIn, which may be an entry to job opportunities and a way to socially link to lecturers as well as their networks. The students are apparently not aware of this possibility, as 80% of the lecturers are not recognized by the students as LinkedIn users and only a few percent of the students are experienced LinkedIn users. Right now, the students at UCPH and many other universities are left to their own assessment of what is relevant for their studies and career and not surprisingly, more than half of the students (53.4%) believe that the IT incorporated in their studies does not provide them with the skills necessary for their future profession. They need guidance from both teachers and the university with respect to how they can use digital services effectively in their studies and what competencies they are required to have in their professional life. Universities cannot expect digital natives to be self-directed learners who are able to navigate on their own without guidance or any other assistance. Therefore, it is important that lecturers align their expectations with those of the students and introduce the students to relevant academic digital services and tools as part of their teaching and learning activities. Moreover, they should consider whether it may be relevant to incorporate new media in their teaching as this digital universe is familiar to the students. This can be accomplished by integrating ICT requirements in the curricula.

Returning to the concept of digital natives, it is important to note that although the term makes them sound both competent and creative in their use of ICT, they have a mediatized approach to materials and how to find knowledge and there are significant differences within the group. Prensky refers to them as digitally divided [10]. What is important is not just basic knowledge of how to use ICT but also digital literacy, which includes the ability to adapt to new challenges and conditions in a rapidly changing digital world. This includes skills in critical information retrieval, data processing and the ability to take advantage of the diversity of digital media.

With this in mind, lecturers and the university need to address this challenge by both meeting the digital literate students with advanced learning technologies as well as offering services that will increase the digital literacy of those in need.

The lecturers need to incorporate ICT in the teaching and learning activities offered, for example, by using more visual media, providing feedback and setting up environments that allow students to develop their mediated approach to knowledge, materials and how to find it. Furthermore, it is the university’s role to nurture the students’ digital literacy, ensuring that the students obtain the necessary knowledge, skills and competencies to master media, information and communication processes.

### Limitations of the study

One limitation which may be of major importance for the interpretation of the results is the relatively low Level of participation. Only approximately every eighth student responded to the questionnaire. As the survey was administered digitally an additional risk is that it is the most digitally literate that responded, and this could have led to an overestimation of the number of students who have computers and smartphones as well as of the level of digital competence and behaviour.. However, since our conclusions lean in the opposite direction, towards the notion that the students may need to be more digitally literate and do not appear to be fully digital natives, this may not be a problem. It should be noted that more than 1100 students from a variety of medical and science-related study programs, at different study levels and of different ages actually did participate.

## Conclusions

Although a large percentage of the students that responded to the survey had access to the internet and basic software skills and used online services in their private lives, they lacked familiarity with the software and services that the lecturers expect them to use in relation to their study activities. The students experienced that teachers did not use digital services such as social media and professional networks or other internet resources, which apparently influenced their perception of their importance and thereby their usage of these services and resources in relation to their studies. The way the younger students communicate differed from the way communication takes place at the university and it is recommended that the university examines how the faculty and the administration can communicate with the students in ways they are familiar with.

## Additional file

Additional file 1: **Questionnaire.** English version of questionnaire administered to students.

## Abbreviations

UCPH: University of Copenhagen
LMS: Learning management system
