# Supplementary material for: Transforming students into digital academics: a challenge at both the individual and the institutional level
Source: BMC Med Educ. 2015 Mar 14;15:48. doi: 10.1186/s12909-015-0330-5 (PMC4377857; doi:10.1186/s12909-015-0330-5)
Supplement: Additional file 1: — Questionnaire. English version of questionnaire administered to students. [file 12909_2015_330_MOESM1_ESM.pdf]

This is a survey concerning the IT-skills at Faculty of Health and Medical Sciences.

You can select language by choosing one of the flags in the bottom of the survey.

What is your current level of education?

(1) ❑ Bachelor

(2) ❑ Part time Master

(3) ❑ Full time Master

At what year are you currently enrolled?

(1) ❑ 1st year

(2) ❑ 2nd year

(3) ❑ 3rd year

(4) ❑ 4th year

(5) ❑ 5th year

(6) ❑ 6th year

1. Which type of mobile phone do you normally use?

(1) ❑ A smartphone

(2) ❑ Not a smartphone

(3) ❑ Do not use a mobile phone

Which kind of smartphone?

(1) ❑ iOS (Apple)

(2) ❑ Android

(3) ❑ Windows

(4) ❑ Other

2. Which type(s) of tablet and/or computer do you normally use?

Do you have one or more of the following devices: Stationary PC/computer, tablet, mobile computer (laptop)?

(1) ❑ Yes

(2) ❑ No, I do not have any of these devices

Do you have a stationary PC/computer?

(1) ❑ Yes

(2) ❑ No

Which kind of operating system do you use on your stationary PC/computer?

(1) ❑ Windows

(2) ❑ OS (Apple)

(3) ❑ Linux

(4) ❑ Others

Do you have a tablet?

(1) ❑ Yes

(2) ❑ No

Which kind?

(1) ❑ iOS (Apple)

(2) ❑ Android

(3) ❑ Windows

(4) ❑ Other

Battery lifetime on your tablet? (Hint: Think about when your battery runs out during an education day)

(1) ❑ 0-2 hours

(2) ❑ 2-4 hours

(3) ❑ 4-6 hours

(4) ❑ 6-8 hours

(5) ☐ 8-10 hours

(6) ☐ 10+ hours

Do you have a mobile computer (laptop)?

(1) ☐ Yes

(2) ☐ No

Which kind of operating system do you use on your mobile computer (laptop)?

(1) ☐ Windows

(2) ☐ OS (Apple)

(3) ☐ Linux

(4) ☐ Others

Battery lifetime on your mobile computer (laptop)? (Hint: Think about when your battery runs out during an education day)

(1) ☐ 0-2 hours

(2) ☐ 2-4 hours

(3) ☐ 4-6 hours

(4) ☐ 6-8 hours

(5) ☐ 8-10 hours

(6) ☐ 10+ hours

3. How do you access the internet? (Multiple answers are allowed)

(1) ❑ By stationary PC/computer

(2) ❑ By mobile phone

(3) ❑ By tablet

(4) ❑ By mobile computer (laptop)

4. How often do you use?

	Less than monthly	Once a month	Several times a month	Once per week	Several times per week	On a daily basis	Do not use
KU's virtual learning system Absalon	(7) ❑	(1) ❑	(2) ❑	(3) ❑	(4) ❑	(5) ❑	(6) ❑
KU mail	(7) ❑	(1) ❑	(2) ❑	(3) ❑	(4) ❑	(5) ❑	(6) ❑
KUnet's other functions, e.g. bulletin board and self service	(7) ❑	(1) ❑	(2) ❑	(3) ❑	(4) ❑	(5) ❑	(6) ❑

5. How often do you use the following platforms in relation to your education?

	Less than monthly	Once a month	Several times a month	Once per week	Several times per week	On a daily basis	Do not use
Social networks e.g. Facebook	(1) ❑	(2) ❑	(3) ❑	(4) ❑	(5) ❑	(6) ❑	(7) ❑
Professional networks e.g. LinkedIn	(1) ❑	(2) ❑	(3) ❑	(4) ❑	(5) ❑	(6) ❑	(7) ❑
Video/sound/images e.g. YouTube or Instagram	(1) ❑	(2) ❑	(3) ❑	(4) ❑	(5) ❑	(6) ❑	(7) ❑
Micro blogging e.g. Twitter	(1) ❑	(2) ❑	(3) ❑	(4) ❑	(5) ❑	(6) ❑	(7) ❑
Reference to online materials e.g. E-journals	(1) ❑	(2) ❑	(3) ❑	(4) ❑	(5) ❑	(6) ❑	(7) ❑
Advanced software e.g. Virtual Microscopy, chemdraw, simulation	(1) ❑	(2) ❑	(3) ❑	(4) ❑	(5) ❑	(6) ❑	(7) ❑

6. How familiar are you with the following?

	1. Not at all	2.	3. To some extent	4.	5. Very much
Word	(1) ❑	(2) ❑	(3) ❑	(4) ❑	(5) ❑

	1. Not at all	2.	3. To some extent	4.	5. Very much
processing e.g. Word					
Spreadsheet e.g. Excel	(1) ❑	(2) ❑	(3) ❑	(4) ❑	(5) ❑
Apps for smartphones	(1) ❑	(2) ❑	(3) ❑	(4) ❑	(5) ❑
Reference management software e.g. EndNote or Reference Manager	(1) ❑	(2) ❑	(3) ❑	(4) ❑	(5) ❑
Redirection of KU mail	(1) ❑	(2) ❑	(3) ❑	(4) ❑	(5) ❑

7. Where do you find information in relation to your assignments? (Multiple answers are allowed)

(1) ❑ Wide search engines e.g. Google

(2) ❑ Narrow search engines e.g. Google Scholar

(3) ❑ Subject-specific e.g. Pubmed

(4) ❑ Library search engines e.g. REX

(5) ❑ None of these

8. Which digital options do you use when working with fellow students? (Multiple answers are allowed)

(1) ❑ Groups

in KUnet

(2) ❑ Projects

in Absalon

(3) ❑ Others

groups e.g. Facebook or LinkedIn

(4) ❑ File

sharing tools e.g. Dropbox, Google drive or Sky drive

(5) ❑ Shared processing of documents e.g. Google Docs

(6) ❑ E-mail

(7) ❑ None of these

9. How do you most often communicate with fellow students? (Hint: By short message, we mean communication between two or more persons, which are not present at the same time. By chat we mean communication between two or more persons present at the same time)

(1) ❑ Short messages e.g. SMS

(2) ❑ E-mail

(3) ❑ Chat e.g. chat in social media

10. If you use a smartphone or tablet, please specify:

(1) ❑ I

have got at least one new app in the last month

(2) ❑ I

have apps that I use only in relation to my education

(3) ❑ I cannot answer yes to any of the above metioned statements

11. To what extent do you get help in relation to new apps?

	1. Never	2.	3. Some	4.	5. Always
Download	(2) ❑	(3) ❑	(1) ❑	(4) ❑	(5) ❑
Settings	(2) ❑	(3) ❑	(1) ❑	(4) ❑	(5) ❑

12. Which of these services do you use outside your education? (Multiple answers are allowed)

(1) ❑ Borger.dk

(2) ❑ Sundhed.dk

(3) ❑ Skat.dk

(4) ❑ News (e.g. New York Times, The Sun, The Wall Street Journal)

(5) ❑ Social media (e.g. Facebook, LinkedIn, Instagram, Youtube)

(6) ❑ None of these

13. How often have you experienced that your teachers have used Absalon in relation with your education?

(Please answer according to your experience during the past 6 months)

(1) ❑ Less than monthly

(2) ❑ Once a month

(3) ❑ Several times a month

(4) ❑ Once per week

(5) ❑ Several times per week

(6) ❑ On a daily basis

(7) ❑ Do not use

14. How often do your teachers use other services than Absalon in relation to your education?

	Less than monthly	Once a month	Several times a month	Once per week	Several times per week	On a daily basis	Do not use
Social networks e.g. Facebook	(1) ❑	(2) ❑	(3) ❑	(4) ❑	(5) ❑	(6) ❑	(7) ❑

	Less than monthly	Once a month	Several times a month	Once per week	Several times per week	On a daily basis	Do not use
Professional networks e.g. LinkedIn	(1) ❑	(2) ❑	(3) ❑	(4) ❑	(5) ❑	(6) ❑	(7) ❑
Video/sound/images e.g. YouTube or Instagram	(1) ❑	(2) ❑	(3) ❑	(4) ❑	(5) ❑	(6) ❑	(7) ❑
Document sharing e.g. Google docs	(1) ❑	(2) ❑	(3) ❑	(4) ❑	(5) ❑	(6) ❑	(7) ❑
Micro blogging e.g. Twitter	(1) ❑	(2) ❑	(3) ❑	(4) ❑	(5) ❑	(6) ❑	(7) ❑
Reference to online materials e.g. E-journals	(1) ❑	(2) ❑	(3) ❑	(4) ❑	(5) ❑	(6) ❑	(7) ❑
Advanced software e.g. Virtual Microscopy, chemdraw, simulation	(1) ❑	(2) ❑	(3) ❑	(4) ❑	(5) ❑	(6) ❑	(7) ❑

15. During your education you are expected to aquire the IT-skills necessary for you to manage future professions. To what extent do you believe that the degree of IT incorporated into your education is sufficient for you to aquire these skills?

(1) ☐ 1. Too little

(2) ☐ 2.

(3) ☐ 3. Appropriate

(4) ☐ 4.

(5) ☐ 5. Too much

Do you have a comment about the use of IT in your education?

____

____

____

____

____

____

____

____

____

____
